# Synchrotron-based characterization of arthroprosthetic CoCrMo particles in human bone marrow

**DOI:** 10.1007/s10856-022-06675-2

**Published:** 2022-06-13

**Authors:** Janosch Schoon, Bernhard Hesse, Remi Tucoulou, Sven Geissler, Melanie Ort, Georg N. Duda, Carsten Perka, Georgi I. Wassilew, Giorgio Perino, Anastasia Rakow

**Affiliations:** 1 Center for Orthopaedics, Trauma Surgery and Rehabilitation Medicine, University Medicine Greifswald, 17475 Greifswald, Germany; 2 Julius Wolff Institute, Berlin Institute of Health at Charité - Universitätsmedizin Berlin, Augustenburger Platz 1, 13353 Berlin, Germany; 3 Xploraytion GmbH, 10625 Berlin, Germany; 4 ESRF-The European Synchrotron, 38000 Grenoble, France; 5 BIH Center for Regenerative Therapies (BCRT), Berlin Institute of Health at Charité - Universitätsmedizin Berlin, Augustenburger Platz 1, 13353 Berlin, Germany; 6 Center for Musculoskeletal Surgery, Charité - Universitätsmedizin Berlin, Charitéplatz 1, 10117 Berlin, Germany

## Abstract

Particles released from cobalt-chromium-molybdenum (CoCrMo) alloys are considered common elicitors of chronic inflammatory adverse effects. There is a lack of data demonstrating particle numbers, size distribution and elemental composition of bone marrow resident particles which would allow for implementation of clinically relevant test strategies in bone marrow models at different degrees of exposure. The aim of this study was to investigate metal particle exposure in human periprosthetic bone marrow of three types of arthroplasty implants. Periprosthetic bone marrow sections from eight patients exposed to CoCrMo particles were analyzed via spatially resolved and synchrotron-based nanoscopic X-ray fluorescence imaging. These analyses revealed lognormal particle size distribution patterns predominantly towards the nanoscale. Analyses of particle numbers and normalization to bone marrow volume and bone marrow cell number indicated particle concentrations of up to 1 × 10^11^ particles/ml bone marrow or 2 × 10^4^ particles/bone marrow cell, respectively. Analyses of elemental ratios of CoCrMo particles showed that particularly the particles’ Co content depends on particle size. The obtained data point towards Co release from arthroprosthetic particles in the course of dealloying and degradation processes of larger particles within periprosthetic bone marrow. This is the first study providing data based on metal particle analyses to be used for future in vitro and in vivo studies of possible toxic effects in human bone marrow following exposure to arthroprosthetic CoCrMo particles of different concentration, size, and elemental composition.

**Figure:**
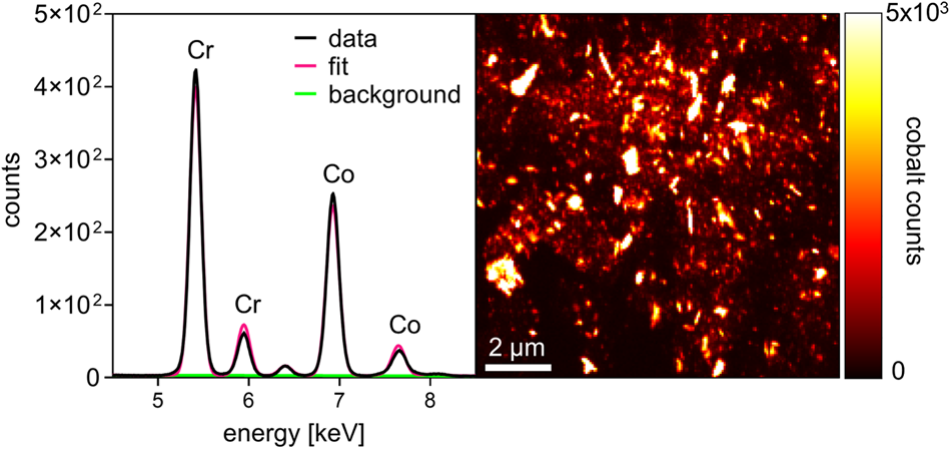
Graphical abstract

Graphical abstract

## Introduction

Cobalt-chromium-molybdenum (CoCrMo) metal alloy is extensively used for medical implants, especially for orthopedic implants. CoCrMo is characterized by favorable mechanical properties such as high mechanical strength, toughness and distinct wear resistance [1, 2]. Also, CoCrMo is seen as being highly biocompatible and therefore widely used for bearing surface and junction components in hip, knee, and shoulder arthroplasty with high load-bearing requirements. However, understanding the reactivity of wear particles released from CoCrMo components in relation to their physicochemical characteristics is one of the key factors for evaluating underlying biological responses [3, 4] and the biocompatibility of wear particles containing CoCrMo. The release of metallic particles and ions has been reported primarily for total hip arthroplasty (THA) and hip resurfacing (HR) implants with bearing components made of CoCrMo [5–7]. Arthroplasty registry studies of metal-on-metal and metal-on-polyethylene hip implants have indicated worse survival rates of some models, as compared to articular bearings made of alternative materials [8, 9]. THA implants with CoCrMo dual modular neck junction (mTHA) have also been associated with formation of particulate and ionic degradation products predominantly by fretting/crevice corrosion [10–12]. In total knee arthroplasty (TKA), CoCrMo is commonly used for the femoral and also often for the tibial components, both of which are paired to a highly crosslinked polyethylene liner. In the late postoperative course, fading of this polyethylene liner can result in direct contact of the metal components leading to significantly increased wear and abrasion rates [13]. Moreover, metal corrosion has also been reported in long stem femoral components at the femoral condyle/stem junction [14].

Numerous factors influence the physicochemical characteristics of wear particles and the associated adverse local tissue reactions (ALTR). They can be classified in two main types: in the periprosthetic soft tissues, predominantly lymphocytic with presence/absence of tissue necrosis or predominantly macrophage component with minimal or no lymphocytic infiltrate; in the bone marrow macrophage infiltrate with or without formation of lymphocytic aggregates [15]. Nanoanalysis of wear particles of three major configurations of hip arthroplasty resulted in a different degree of cell necrosis and distribution of inflammatory infiltrate in the periprosthetic soft tissues [16]. Thus, exposure to these non-engineered particles is of clinical relevance since there is evidence that ALTRs lead to early implant revision and to higher rates of a second revision due to increased risks of aseptic loosening [17] and possibly periprosthetic infections [18].

In a recent study, we demonstrated that particles from primary and revision arthroplasty implants are present in periprosthetic bone marrow, i.e., adjacent to the periprosthetic membrane which had been thought to serve as a bone marrow-isolating barrier [13]. Analyses of the potential of submicron- and nanoparticles to cross cellular and matrix barriers are relevant for the evaluation of possible local as well as systemic toxic effects. Generally, the difference between metal exposure due to particle release from orthopedic implants compared to exogenous exposure scenarios is that a first natural line of cellular defense according to the route of exposure is missing, with possible exposure of internal organs to variable amounts of complex metallic wear debris. Histopathological examinations of the periprosthetic membrane reveal that another important characteristic of this endogenous exposure scenario is that there is a large variability in terms of CoCrMo-derived particles’ physicochemical properties, such as shape, size and elemental composition [19]. Notably, endogenously released particles from arthroplasty implants are not chemically stabilized by e.g., polymer coating as applied for engineered particles [20, 21]. The comparison of elemental ratios of CoCrMo-derived particles with the composition of the implanted bulk alloy allows for drawing conclusions about the particles’ fate in terms of dealloying. Microscale particles in human periprosthetic bone marrow released from arthroplasty implants in vivo show a lower Co content compared to the bulk alloy [13]. The ratios of the alloying metals in bone marrow resident particles as a function of particle size have not yet been investigated. Together with an evaluation of the bone marrow specific particle dosimetry (particle number/bone marrow cell number), the information on the particles’ potential to release ions is of distinct importance for future investigations of particle toxicity in the context of regulatory investigations such as the in vitro or in vivo simulations of wear and corrosion processes and the subsequent characterization of released particles with respect to size distribution. The toxicity of metal-containing micro- and nanoparticles is determined by exposure level and duration, their physicochemical properties, and by their passive and cell-mediated degradation potential, i.e., their potential for dealloying processes hence ion release [22, 23]. In particular, studying particle numbers and characteristics of particles in bone marrow is relevant from a toxicological perspective, considering the high proliferative potential and the diverse cell composition of the hematopoietic marrow.

This work focused on drawing conclusions regarding wear particle processing and degradation in the human bone marrow to learn more about the toxic potential of CoCrMo-derived particles. Primary aim of this study was to analyze CoCrMo-containing micro-, submicron-, and nanoparticles across different arthroplasty implants in human periprosthetic bone marrow in terms of size distribution and elemental ratios with respect to particle size by synchrotron-based nano-X-ray fluorescence (nano-XRF). Secondary aim was to derive a particle dosimetry specific for human periprosthetic bone marrow. These analyses will help to perform future in vitro and in vivo evaluations of the particles’ potential to induce clinically relevant adverse effects following exposure to realistic numbers and well characterized particles from arthroplasty implants. Generally, such approaches may help to support risk-benefit evaluations of the CoCrMo alloy used in orthopedics all over the world on a daily basis.

## Materials and methods

### Patient and implant data

**Fig. 1:**
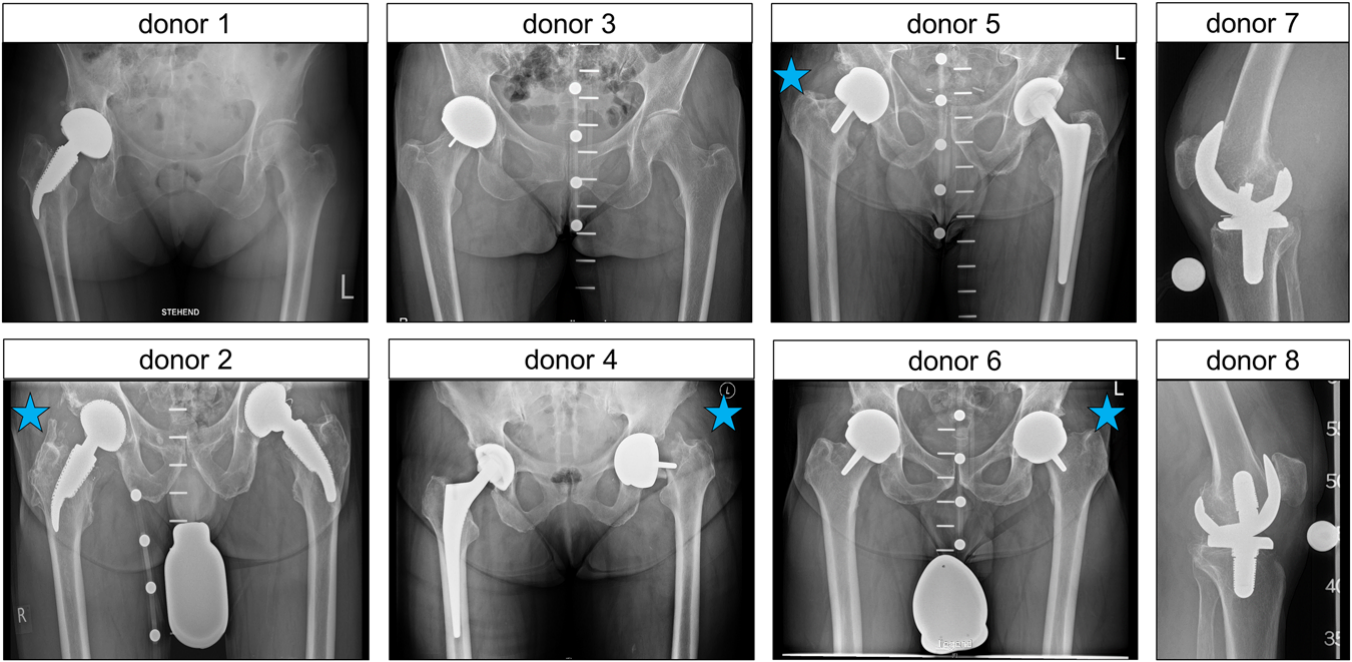
Radiographs taken before index revision surgery, i.e., bone marrow sample extraction (donors 1–6, low-centered ap pelvis; donors 7 & 8, lateral knee view). Blue asterisks indicate the respective index joint. Donor 1–2, modular short stem total hip arthroplasty; donor 3–6, hip resurfacing implant; donor 7–8, total knee arthroplasty

Periprosthetic bone marrow from eight patients with different loosened hip and knee implants were analyzed. Two females and two males undergoing revision of a HR implant were included. At revision surgery, these patients were between 60 and 78 years of age, and their primary HR dated back between 11 and 14 years. The mTHA group comprised a 61-year-old female and a 73-year-old male who underwent re-revision of a modular short stem THA 11 years after acetabular cup revision and 12 years after THA revision, respectively. Further, samples were taken from two males aged 71 and 75 years, respectively, who underwent revision TKA, 19 and 16 years after primary TKA, respectively. Characteristics of patients undergoing revision arthroplasty including implant data (Table 1) and radiographs before index revision surgery (Fig. 1) are reported.

**Table 1:** Patient and implant data

Donor	Age	Sex	Index implant category	Indication for revision surgery	Arthroplasty implant history at index joint	Years since primary arthroplasty
1	61	w	mTHA	Recurrent dislocation, implant loosening	Primary implant: modular short stem THA (ESKA, CUT), non-cemented; 3 years after primary implantation: cup revision to non-cemented TMT press-fit cup, primary modular short stem retained	14
2	73	m	mTHA	Arthroprosthetic metal release, pseudotumor	Primary implant: THA (ESKA, CUT), non-cemented; 4 years after primary implantation: revision to modular short stem THA due to fracture of ceramic head	16
3	63	w	HR	Arthroprosthetic metal release, pseudotumor, elevated systemic metal levels, central acetabular defect	Primary implant in situ: McMinn HR	12
4	78	w	HR	Periprosthetic femoral fracture secondary to osteolysis of the femoral neck	Primary implant in situ: McMinn HR	11
5	78	m	HR	Arthroprosthetic metal release, progressive pain, acetabular and femoral osteolyses	Primary implant in situ: Cormet HR	14
6	60	m	HR	Progressive pain, acetabular and femoral osteolyses	Primary implant in situ: McMinn HR	12
7	75	m	TKA	Arthroprosthetic metal release, progressive pain, PE inlay abraded, synovitis, pseudotumor	Primary implant in situ: TKA	16
8	71	m	TKA	Arthroprosthetic metal release, UHMWPE inlay abraded, implant loosening, femoral and tibial osteolyses	Primary implant in situ: TKA	19

*HR* hip resurfacing, *mTHA* modular short stem THA, *PE* polyethylene, *THA* total hip arthroplasty, *TKA* total knee arthroplasty, *TMT* trabecular metal technology, *UHMWPE* ultra-high-molecular-weight polyethylene

Specimens from nine patients (four females, five males, aged between 44 and 77 years at sampling) undergoing primary arthroplasty were sampled for cell number determination.

### Sample harvest

During revision arthroplasty, a variable amount of periprosthetic bone marrow is routinely debrided from the implant bed. These bone samples were prepared for subsequent nano-XRF analyzes. Implant-material-naïve bone marrow samples were harvested from the medullary canal of the femur which is routinely accessed in the course of primary hip arthroplasty. Ethics approvals were obtained from the local ethics committees of the University Medicine Greifswald (BB 178/20) and the Charité - University Medicine Berlin (EA1/194/13) in accordance with the World Medical Association Declaration of Helsinki.

### Preparation of periprosthetic samples

Details on sample preparation for subsequent nano-XRF analyses were described previously [13]. In brief, bone marrow samples were fixed in paraformaldehyde solution and embedded in poly(methyl methacrylate) (Technovit 9100, Kulzer) according to the manufacturer’s instructions. 10 µm thin sections were prepared with a tungsten carbide blade for the subsequent XRF analyses. For orientation purposes, the section adjacent to the section used for nano-XRF imaging was stained with hematoxylin and eosin.

### Nano-XRF scanning

Data were collected at beamline ID16B of the European Synchrotron Radiation Facility (ESRF) [24]. Details on the settings were described elsewhere [13, 25]. In brief, a pink beam of a center energy of 25.6 keV was selected and focused to 60 × 60 nm spot size. The sample sections were mounted and scanned at 1 µm step width to obtain overviews for further selection of regions of interest to be scanned at 60 nm step width. For each position XRF spectra were collected using an SDD detector system and de-convoluted using the software PyMCA [26].

### Isolation of bone marrow mononuclear cells and cell counting

BM-MNCs were isolated from implant-material-naïve human bone marrow by density gradient centrifugation using at a density of 1.077 g/cm^3^ and 400 × *g* as described previously [13]. Total cell number was counted using the Neubauer approach. Cell numbers were than normalized to the total sample volume assuming a bone marrow density of 1.0 g/cm^3^.

### Image data processing and statistics

**Fig. 2:**
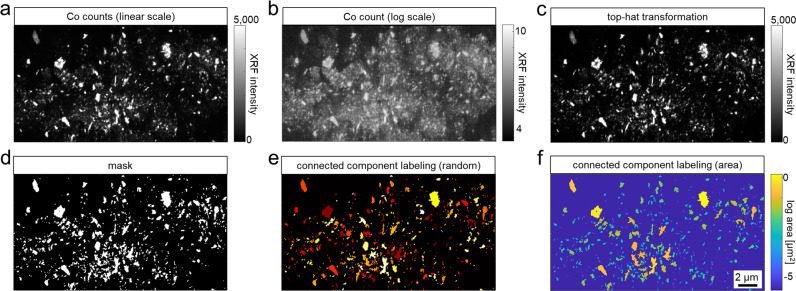
Representative workflow for data processing of the nano-XRF map of periprosthetic bone marrow from a patient with hip resurfacing implant. **a**, **b** Linear scale and log scale maps of the spatial Co signals. **c** Linear scale map of the spatial Co signal following top-hat transformation. **d** Map indicating all signals equal and greater than the derived Co signal threshold. **e** Connected component labeling indicating all particles included for the subsequent analyses of (**f**) particle size, number and elemental composition

The Co intensity map (Fig. 2a, b) was used for input for a subsequent top-hat transformation with a disk-shaped structuring element with a radius of 6 pixel (Fig. 2c), a mask was obtained through thresholding of the top-hat transformed Co maps. (Fig. 2d). The threshold was selected manually such that all map-individual minimum amplitude Co signals were allocated to the Co mask for further analyzes. The Co masks were used as input for the subsequent connected component analysis for each of 60nm-step width maps (Fig. 2e, f). The connected component analysis was coupled to the intensity maps of Co, Cr and Mo to obtain the area and the mean intensity of Co, Cr and Mo for each particle. Approximating that the analyzed particles consist of spherical and non-hollow spheres, the particle area was used for calculating the particles’ diameter.

### Data processing and statistics

Descriptive statistics regarding RGB imaging and peak spectra was realized with PyMCA [26]. ImageJ was used for the thresholding of the Co-maps. Top-hat transformation of the Co-maps, mask generation, connected component analyses and element specific intensity quantification of particles were conducted by Matlab R2021a using the Image Processing Toolbox. Data plotting and explorative statistics was implemented by GraphPad Prism 8. No samples or data were excluded from the analyses.

## Results

### Metal exposure in periprosthetic bone marrow

**Fig. 3:**
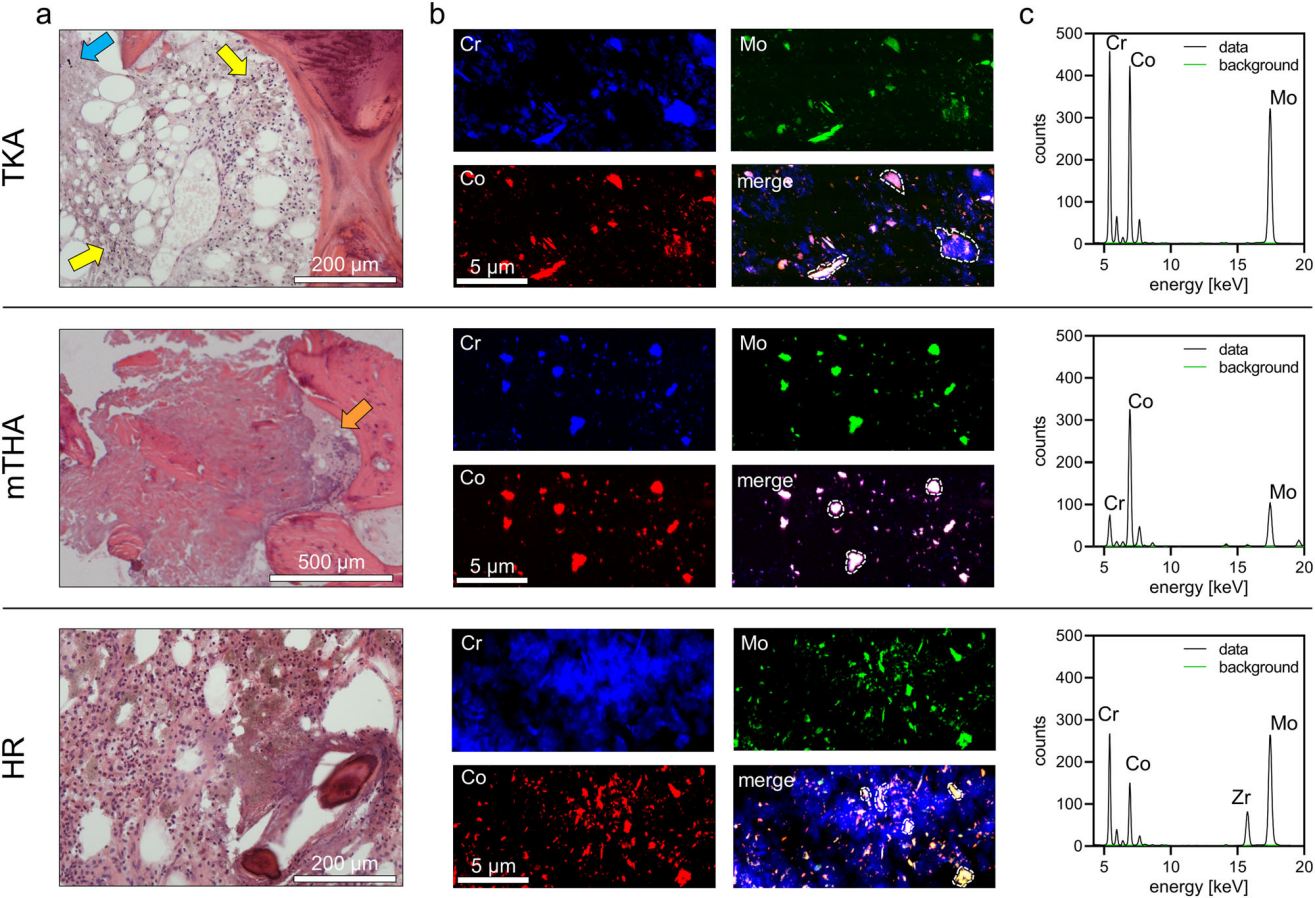
Exposure to CoCrMo particles in periprosthetic bone marrow samples from three patients undergoing revision surgery of a total knee arthroplasty implant (TKA), a modular hip arthroplasty implant with modular femoral component (mTHA) and a hip resurfacing arthroplasty implant (HR). **a** Histopathological evaluations of hematoxylin and eosin stained sections (consecutive sections of the sections analyzed by XRF) revealed presence of micron sized particles (blue arrow) in an area of necrotic macrophages and submicron-particles and particle aggregates in areas with clusters of particle-laden macrophages (yellow arrows) (TKA), micron sized particles in a large area of cell necrosis adjacent to an area of dystrophic calcification (orange arrow) (mTHA) and infiltration of particle-laden macrophages in an area with presence of non-interconnected bone trabeculae (HR). **b** RGB imaging of the spatially resolved metal specific XRF signals at a step size of 60 nm indicates abundance of CoCrMo containing particles. **c** XRF-spectra of micron sized particles (dashed lines, see **b**) clearly demonstrate that these particles consist of Co, Cr and Mo.

Sections of periprosthetic human bone samples were prepared and histologically processed for histopathological evaluation and orientation purpose in the course of nano-XRF scanning. Histopathology revealed the presence of a variable degree of macrophage infiltration with clusters of cell necrosis (Fig. 3a). On the adjacent unstained sections, spatially resolved multi-element analyses were performed using synchrotron-based nano-XRF analyses (Fig. 3b). Nano-XRF analyses at a step size of 60 nm revealed substantial exposure to Co, Cr and Mo containing particles in 10 nano-XRF maps of bone marrow adjacent to TKA, mTHA and HR implants (Fig. 3c). These maps from periprosthetic bone marrow samples of three different implant types were divided accordingly: TKA, 3 maps from 3 bone marrow samples from 2 patients; mTHA, 2 maps from 2 bone marrow samples from 2 patients; HR, 5 maps from 5 bone marrow samples from 4 patients.

In summary, the nano-XRF analyses revealed a detectable presence of CoCrMo particles in the periprosthetic bone marrow in all cases of TKA, mTHA and HR implants.

### Particle size distribution

Analyses of particle size frequency distributions of CoCrMo particles released from three different implant types into the bone marrow were performed following data processing including thresholding of all individual top-hat transformed Co maps.

**Fig. 4:**
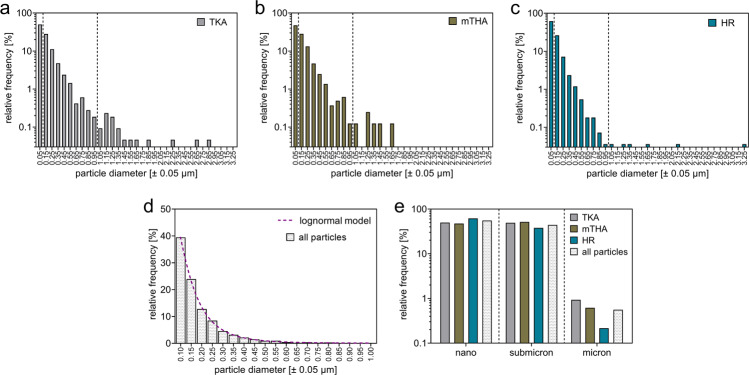
Size frequency evaluation of particles from three different implant types revealed distribution pattern towards more frequent occurrence of particles in the nano- and submicron-scale. **a**–**c** Particle size distribution in periprosthetic bone marrow samples proximate to total knee arthroplasty implants (TKA), modular total hip arthroplasty implants (mTHA) and hip resurfacing implants (HR). **d** Particle size distribution of all analyzed particles follows the lognormal model. **e** Separation of frequencies of particle sizes into different size ranges indicates the lowest proportion of microparticles in the HR group

These analyzes of particles in the bone marrow from the proximity of TKA, mTHA and HR implants, revealed frequency distributions indicating a decreasing fraction as a function of increasing particle size (Fig. 4a–c). Overall, large part of the detected particles was in the nanoscale (<100 nm) and submicron scale (≥100 nm–<1 µm). The particle size frequencies of all analyzed particles within this range can be described with lognormal particle size distribution (*R*^*2*^ = 0.9992) (Fig. 4d). The overall frequency of all identified particles of micron scale (≥1 µm) was 0.56%. These microparticles were predominantly found in the bone marrow adjacent to TKA and mTHA implants with frequencies of 0.93 and 0.62%, respectively. In contrast, only 0.22% of all particles in the HR group could be assigned to the micron scale (Fig. 4e).

Taken together, the analyses of particle sizes showed lognormal particle size distribution patterns predominantly towards the nanoscale.

### Particle numbers in periprosthetic bone marrow

To assess the bone marrow concentrations of detected particles and particles of different size ranges, the particles were counted and normalized to the analyzed volume of the individual bone marrow section and the individually analyzed area, respectively (Table 2).

**Table 2:** Numbers of nano-, submicron-, and microparticles related to the sample specific bone marrow volume and related to cell counts of native bone marrow samples to approximate particle numbers per bone marrow cell

	Number of particles identified	V map [ml × 10^−9^]	Particles/ml BM [×10^9^]	Particles/BM cell [min. to max.]
Total knee arthroplasty - nanoparticles				
Min. particle exposure	35	5.3	6.6	143–991
Max. particle exposure	696	4.1	171.3	3697–25,570
Sum of all regions analyzed	1069	12.1	88.5	1902–13,218
Modular total hip arthroplasty - nanoparticles				
Min. particle exposure	6	2.3	32.5	57–394
Max. particle exposure	378	11.7	2.6	697–4846
Sum of all regions analyzed	384	13.9	27.6	593–4118
Hip resurfacing - nanoparticles				
Min. particle exposure	22	2.0	10.8	213–1609
Max. particle exposure	952	6.8	139.5	2997–20,827
Sum of all regions analyzed	1711	16.4	104.1	2237–15,546
All implant types - nanoparticles				
Sum of all regions analyzed	3164	42.4	74.6	1602–11,134
Total knee arthroplasty - submicron sized particles				
Min. particle exposure	47	5.3	8.9	192–1331
Max. particle exposure	631	4.1	155.3	3335–23,182
Sum of all regions analyzed	1057	12.1	87.5	1880–13,069
Modular total hip arthroplasty - submicron sized particles				
Min. particle exposure	23	2.3	10.1	217–1509
Max. particle exposure	395	11.7	33.9	729–5064
Sum of all regions analyzed	418	13.9	30.0	645–4483
Hip resurfacing - submicron sized particles				
Min. particle exposure	25	2.0	12.2	263–1828
Max. particle exposure	397	2.7	149.8	3219–22,373
Sum of all regions analyzed	1049	16.4	63.8	1371–9531
All implant types - submicron sized particles				
Sum of all regions analyzed	2524	42.4	59.5	1278–8882
Total knee arthroplasty - micron sized particles				
Min. particle exposure	0	5.3	0	0
Max. particle exposure	12	4.1	3.0	63–441
Sum of all regions analyzed	20	12.1	1.7	36–247
Modular total hip arthroplasty - micron sized particles				
Min. particle exposure	5	11.7	0.4	9–64
Max. particle exposure	1	2.3	0.4	9–66
Sum of all regions analyzed	6	13.9	0.4	9–64
Hip resurfacing - micron sized particles				
Min. particle exposure	0	6.8	0	0
Max. particle exposure	4	2.0	2.0	42–292
Sum of all regions analyzed	6	16.4	0.4	8–55
All implant types - micron sized particles				
Sum of all regions analyzed	32	42.4	0.75	16–113

*BM* bone marrow

To estimate the entire range, i.e., the min. and max. particle number/volume, the region with the lowest and the region with the highest particle number/volume were selected for each implant class. Furthermore, the total volumes of all regions were calculated and the sum of all detected particles was normalized to this volume. The highest nanoparticle concentration was found in the surrounding bone marrow of the HR group (104.11 × 10^9^ nanoparticles/ml bone marrow). In contrast, the highest concentration of submicron- and microparticles was detected in the bone marrow of the TKA group (87.53 × 10^9^ submicron particles/ml bone marrow and 1.66 × 10^9^ microparticles/ml bone marrow). In addition, cell counts of bone marrow mononuclear cells (BM-MNCs) of nine native (implant-material-naïve) metaphyseal bone marrow samples were determined to derive an approximation of the spatial particle density to the spatial density of bone marrow cells. Cell counting revealed a median cell number of 2.66 × 10^7^ BM-MNCs/ml bone marrow and a range of 6.70 × 10^6^–4.65 × 10^7^ BM-MNCs/ml bone marrow. The minimum and maximum cell concentrations were used to calculate the minimum and maximum particle number/bone marrow cell for the individual region and sum region of the respective implant group.

From this data, a periprosthetic bone marrow specific particle dosimetry was derived for future in vitro and in vivo experiments aiming at emulating exposure to CoCrMo particles in periprosthetic cancellous bone and bone marrow at relevant concentrations. The following order of magnitude is suggested to cover relevant particle numbers/bone marrow cell [min. - max.]: nanoparticles/cell, 1 × 10^1^–1 × 10^5^; submicron particles/cell, 1 × 10^1^–1 × 10^5^; microparticles/cell, 0–1 × 10^3^.

### Composition of particles with respect to particle size

To analyze the bone marrow located CoCrMo particle composition as a function of particle size and thus to be able to draw conclusions about the elemental particle composition at initial release or fate after release, all identified particles were analyzed regarding their mean Co, Cr and Mo counts, their respective elemental ratios, and their respective sizes.

**Fig. 5:**
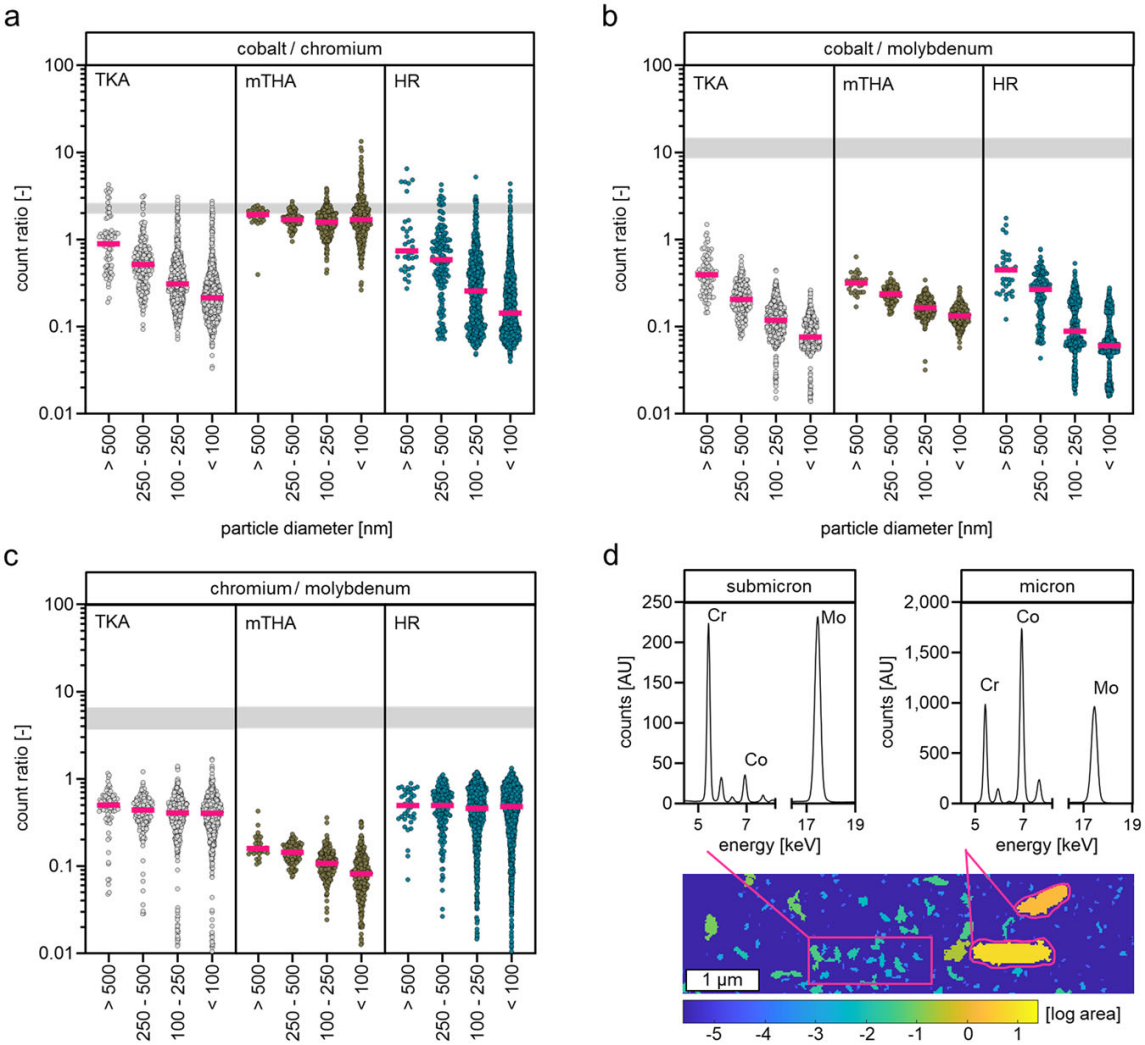
Analysis of the elemental ratios of the CoCrMo particles located in the bone marrow revealed distinct differences of particle compositions with respect to particle sizes. **a** Particles released from total knee arthroplasty implants (TKA) and hip resurfacing implants (HR) indicate a distinct particle size-dependent decrease of Co to Cr ratios. Gray bar indicates the Co to Cr ratio in commonly used CoCrMo alloy (2.0–2.5). **b** Size-dependent Co to Mo ratio in particles released from all analyzed implant types. Gray bar indicates the Co to Mo ratio in commonly used CoCrMo alloy (8.6–14.7). **c** The Cr to Mo ratio is not influenced by particle size in particles released from TKA and HR implants while particles from mTHA implants are characterized by a size-dependent decrease of the Cr to Mo ratio. Gray bar indicates the Cr to Mo ratio in commonly used CoCrMo alloy (3.7–6.7). **d** Comparison of the XRF-spectra of submicron sized particles (left) and micron sized particles (right) released from a TKA implant clearly demonstrate that smaller particles are characterized by a lower Co content in relation to Cr and Mo

The analyses showed an altered Co to Cr ratio as a function of particle size for particles released from TKA and HR implants (Fig. 5a). Smaller CoCrMo particles are characterized by a lower Co content in relation to the Cr content. Nanoparticles (≤100 nm) show a 4.2-fold lower median Co to Cr ratio (TKA) and a 5.2-fold lower median Co to Cr ratio (HR) than the respective microparticles (≥0.5 µm). In contrast, CoCrMo particles released from mTHA implants do not show these distinct differences in the Co to Cr ratio, indicated by an only 1.2-fold lower median Co to Cr ratio of nanoparticles compared to microparticles. In contrast to released microparticles, submicron- and nano-sized particles have a distinctively lower Co to Cr ratio than CoCrMo bulk alloy. In order to evaluate whether a lower Co to Cr ratio of smaller particles is associated to a lower Co content, Co counts of the particles were additionally related to the respective counts of the oxidatively stable Mo (Fig. 5b). The analyses clearly show that the Co to Mo ratio also strongly depends on particle size. Smaller CoCrMo particles released from all analyzed implant types are characterized by a lower Co content in relation to the Mo content. Fold changes of the median Co to Mo ratio of microparticles and nanoparticles: TKA (5.2); mTHA (2.4); HR (7.4). The ratio of Co to Mo in submicron- and nano-sized particles as well as in microparticles is distinctively lower than in bulk alloy. This finding additionally indicates that particles at the time of release are characterized by a low Co content or that a pronounced release of Co from CoCrMo particles occurs after release in the bone marrow.

Furthermore, the Cr to Mo ratio of the CoCrMo particles was analyzed (Fig. 5c). The analyses show that the Cr to Mo ratio of particles released from TKA and HR implants is not related to particle size. In contrast, particles released from mTHA implants show a size-dependent decrease in the Cr to Mo ratio; smaller particles have a lower Cr content. In addition, the Cr to Mo ratios of particles of all sizes released from mTHA implants are clearly lower than the ratios of particles released from TKA and HR implants. Overall, the Co and Cr to Mo ratios indicate that the Co and Cr content of CoCrMo particles released from all analyzed implant types is significantly lower than in bulk CoCrMo alloy.

In summary, the elemental ratios of particles released from arthroplasty implants strongly dependent on particle size, with nanoparticles and submicron-sized particles having a significantly lower Co content than microparticles (Fig. 5d). In cases of particle release from mTHA implants, this relationship is also evident for Cr.

## Discussion

The aims of this study were to analyze CoCrMo-containing micro-, submicron-, and nanoparticles across different arthroplasty implant types in human periprosthetic bone marrow with regard to their size, number and elemental composition. The nano-XRF analyzes of the bone marrow samples revealed lognormal frequency distributions of particle sizes, particle numbers that will allow for deriving a particle dosimetry for future in vitro and in vivo experiments, and that particularly the Co content strongly depends on the particles size indicating a low Co content at the time of particle release or Co release from CoCrMo particles within the bone marrow.

Considering the high proportion of bone marrow-resident macrophages and its highly proliferative function ensuring constant releases of mature immune cells into the circulation, the human bone marrow as a periprosthetic organ is of utmost relevance with regard to possible toxic effects of CoCrMo particles [27, 28]. Arthroplasty implants are anchored with direct contact to the bone marrow and it is suggested that this organ plays a vital role in periprosthetic inflammation-associated effects, either following direct exposure or secondary due to macrophage migration from the neo-synovial membrane [29, 30]. The clinically most relevant type of ALTR due to polyethylene and/or metallic particles is periprosthetic osteolysis, a macrophage-induced pathology affecting the quality of trabecular bone which is in direct contact with the bone marrow. Regarding the histopathological examination of particle induced periprosthetic aseptic inflammation, the focus has been on the study of the synovium-like interface membrane, inferring that the results could be also applied to changes in bone homeostasis [15, 31]. However, observations of the periprosthetic bone do not support some of the findings, raising the challenging question whether the macrophage infiltrate in the bone marrow might have a biological behavior which differs from the one observed in the neo-synovial membrane. For this reason, we have recently investigated different manifestations of immune responses in the bone marrow, and found a variable degree of macrophage infiltration/proliferation depending on multiple factors and in some cases associated with a lymphocytic infiltrate with or without the presence of germinal centers [30].

Data regarding size distributions and shapes of arthroprosthetic particles in the bone marrow are not available. In contrast, particle sizes and shapes in neo-synovial interface membrane have been thoroughly described [16, 32, 33]. Ranges of particle sizes in this neo-synovial interface membrane are wide and largely depend on the generating modalities. Generally, it is challenging to categorize particles into sub-groups regarding their origin in terms of specific release mechanisms. At conventional light microscopy examination, metallic wear particles can be broadly classified into two main categories: (1) “conventional particles” generated by abrasion/adhesion/erosion which are usually black due to a variable component of titanium, with the exception of CoCrMo particles generated by edge loading which can turn greenish during the hematoxylin-eosin staining, and (2) “corrosion particles” generated by tribocorrosion at the bearing surface and/or predominantly fretting/crevice corrosion at the junction surfaces, usually greenish/yellowish bound into lysosomal membranes [33]. Conventional membrane located CoCrMo particles show a size distribution pattern from <30 nm to the microscale and infrequently up to the macroscale (>10 µm) [33]. In contrast, particles generated by corrosive processes were found to be mostly in the nanoscale and have the potential to form large nanoparticle agglomerates/aggregates in the neo-synovial membrane and in the interstitial tissue [15, 33]. In the presented study, focusing on particle analyses in the periprosthetic bone marrow, the particle size distribution analyses likewise revealed a broad range of particle sizes from the nano- to the macroscale. Size distribution analyses of particles released from the different implant types show a decreasing fraction with increasing particle size, and the cumulative distribution of the analyzed particles follow a lognormal function. This distribution pattern indicates a natural system assuming normally distributed times for formation or breakdown of particles [34]. In vivo neoformation of particles containing all three alloying metals is unlikely. Analyses of the characteristics of particles from cell-free simulator studies and of particles generated in vivo, indicate that comparative size distribution patterns already occur during particle release [35]. Moreover, cell-mediated particle breakdown in the synovial fluid and periprosthetic tissue or passive i.e., non-cellular mediated breakdown by oxidative dissolution processes could lead to the wide particle size spectrum. To the best of our knowledge, time-resolved studies on the breakdown of CoCrMo particles in biological systems have not been reported. From the particle analyses shown, we learn that sizewise all categories of particles are not only abundant in the neo-synovial membrane but also in the periprosthetic bone marrow. According to histopathological observations, particle exposure in the bone marrow most likely depends on the amount of macrophage infiltrate from the neo-synovium, although wear particles might also migrate directly to the bone marrow pushed by the pressure of the synovial fluid, in particular in cases of formation of large agglomerates/aggregates by fretting/crevice corrosion. Particle-laden macrophages can infiltrate the bone marrow [30] potentially through ameboid movement from neo-synovial membrane. However, the question of whether the particles enter the bone marrow through a macrophage-mediated manner and/or directly cannot be answered by the analyses shown here.

HR implants are known to release wear particles at the bearing surface by tribocorrosion, predominantly associated with the generation of nanoparticles with low Co content, and edge loading, predominantly associated with the generation of nanoparticles and submicron sized particles with higher Co content [36, 37], whereas TKA implants with degeneration and delamination of the polyethylene insert are associated with particle release due to abrasion/erosion/adhesion between the femoral and tibial components [38]. The dissimilar mechanisms of particle release from HR and TKA implants could be the basis for the observed differences in bone marrow concentrations of particles within different size ranges. The performed analyses suggest that concentrations of nanoparticles are higher in bone marrow adjacent to HR implants compared to bone marrow adjacent to TKA implants. In contrast, a higher concentration of micron sized particles was detected in the periprosthetic bone marrow of TKA implants. Modular junctions at the femoral component of hip arthroplasty implants pairing titanium and CoCrMo components are known to release particles mainly due to crevice/fretting corrosion [39], although the femoral component of the analyzed mTHA consists of a CoCrMo dual modular neck and stem [40]. To our knowledge, there is no information on the size, shape and the elemental composition of wear particles released from this dual modular femoral neck hip prosthesis (ESKA CUT 2000) available. For this particular implant design, a cantilever effect and a relatively large contact area potentially result in particle release due to mechanically assisted corrosion, as shown in hip implants with a similar configuration [41].

The particle numbers of up to 100 × 10^9^ particles/ml bone marrow indicate that these numbers are conceivable if compared to particle number magnitudes of those detected in the periprosthetic neo-synovium. Doorn et. al derived a total release of 6.7 × 10^12^–2.5 × 10^14^ CoCrMo particles/year by electron microscopy analyses of periprosthetic tissue from patients with first generation metal-on-metal THA implant [32]. However, systematic quantification of CoCrMo particle numbers from different implant failure mechanisms in ex vivo periprosthetic neo-synovial membrane and synovial fluid has, to the best of our knowledge, not yet been reported. This makes deriving a clinically relevant particle dosimetry for testing particulate degradation products from orthopedic implant materials challenging. The particle counts we now identified in bone marrow, indicate that bone marrow is likewise exposed to particles released from arthroprosthetic implants. The CoCrMo particle numbers and sizes identified, are also important in order to be able to use these as guiding numbers when deriving a particle dosimetry for in vivo experiments in the future. Recently, in response to the updated European medical device regulation (EU MDR), medical device manufacturers together with scientific consultancies published an assessment of the carcinogenicity of CoCrMo-containing medical devices based on in vivo studies [42]. The authors identified two in vivo studies on CoCrMo particles to be highly relevant for the assessment of a risk for local as well as systemic onset of cancer [43, 44]. In these studies, particles with a size of 1.5–50 µm (20 mg/rat) [43] and <3 µm (67 µg/rat) [44] were applied intraarticularly. Considering sizes and quantities of the particles used and the administration regime (single dose), further studies implementing a clinically relevant particle dosimetry and the particle composition are indicated to assess the carcinogenic hazard of CoCrMo particles.

It has been reported that the toxicity of metallic particles depends on the characteristics of their protein corona complex and their potential to release metal ions [45–47]. Metal ion release at the time of particle release occurs largely in the course of tribocorrosion, a degradation mechanism associated with the release of nanoparticles. In vitro corrosion versus tribocorrosion testing revealed that Co dissolution is primarily present under tribocorrosive conditions [48] indicating that the underlying mechanism of particle release strongly influences the particles’ Co content. A further complementary reason for lower Co to Cr ratio of particles compared to bulk CoCrMo is the dissolution of Co within periprosthetic tissues and fluid subsequent to particle release. Smaller particles are characterized by a higher surface-area-to-volume ratio, which leads to a greater potential for ion release resulting from more available surface area [49]. Koronfel et al. were able to show that CoCrMo particles released into periprosthetic tissues are degraded by passive dissolution processes and suggest a dealloying-like mechanism with Co being the predominantly dissolved alloy constitute [3] particularly after breakdown of a Cr-rich passivation film [50]. The correlation of particle sizes and Co to Cr and Co to Mo ratios shown in the present study suggests that Co ion release also occurs in the periprosthetic bone marrow. Co ions are known to have the potential for sensitization [51, 52]. Even though lymphocytic reactions in the periprosthetic neo-synovium have been attributed to metal hypersensitivity in TKA [53, 54], preoperative allergy tests have so far failed to predict clinical signs and symptoms of hypersensitivity [55, 56]. This discrepancy most likely arises from the fact that a cutaneous patch test or lymphocyte transformation test cannot represent the local capacity for a corresponding immune response. This underlines the importance of considering the bone marrow in terms of capacity for specific immune responses to particles from orthopedic implants.

A limitation of our study is that different mechanisms of particle release can occur at different post-operative time intervals, and also at different rate. It is reasonable to assume that wear particles from edge loading (e.g., HR group) and particles from abrasion/erosion/adhesion (e.g., TKA group) occur significantly later than particles from tribocorrosion (e.g., mTHA and HR group). The local persistency of particles could lead to a lowering of the Co and Cr content due to steady dissolution processes. Moreover, it must be considered that the analyses shown here are based on a single, cross sectional observation at the time of revision surgery. Time-resolved particle analyses are suggested to be implemented in animal models or in relevant in vitro bone and bone marrow models [57]. The approximated particle numbers are worst-case numbers because nano-XRF only allows for analyzing small volumes and additionally these analyzes were performed in focal hotspots of particle exposure. In addition, it should be considered that microparticles per se can generate more XRF signal than nanoparticles, since although the X-ray beam is only 60 nm wide (full width at half maximum) the edges of the X-ray beam can generate additional excitation. Therefore, only relative elemental contents were discussed. The ratio of Co to Cr as a function of particle size is also influenced by self-absorption effects, which were not considered in our analysis. However, this effect can be neglected for submicron particles and for the largest particles considered in this study (~3 µm). We estimate that self-absorption effects lead to an underestimation of the Co to Cr ratio of less than 10%. Since we overserved a trend towards smaller Co to Cr ratios with respect to decreasing particle size, the impact of self-absorption is not in conflict with our observations.

## Conclusion

The characterization of CoCrMo particles in the periprosthetic bone marrow of patients undergoing revision surgery of a selection of HR, TKA or mTHA implants revealed a lognormal size distribution dominated by nanoparticles, a particle number of up to 100 × 10^9^ particles/ml bone marrow and a clear correlation of particle size and elemental ratios. These findings point toward a lower Co content of smaller particles at the time of release or increased Co release from particles with respect to decreasing particle size. Due to the pronounced relevance with regard to possible local and systemic toxic effects of particle exposure, the bone marrow per se and the administration of clinically relevant particle numbers, sizes and compositions should be considered in future in vitro and in vivo studies in the context of analyzing biological consequences of arthroprosthetic particle exposure. The data shown here may support future assessment of biological consequences resulting from exposure to CoCrMo particles, in particular the evaluation of the toxicity and carcinogenicity of metallic Co which is classified as a category 1B carcinogen.
